# A Promising Recycling Strategy via Processing Polypropylene/Recycled Poly(ethylene terephthalate): Reactive Extrusion Using Dual Compatibilizers

**DOI:** 10.3390/polym16172439

**Published:** 2024-08-28

**Authors:** Fatemeh Morshedi Dehaghi, Mohammad Aberoumand, Uttandaraman Sundararaj

**Affiliations:** Department of Chemical and Petroleum Engineering, University of Calgary, Calgary, AB T2L1Y6, Canada

**Keywords:** recycling waste plastics, reactive extrusion, in situ graft copolymer, compatibilization, interfacial adhesion, mechanical properties, reactive blending

## Abstract

Enhancing interfacial adhesion in polypropylene (PP)/recycled polyethylene terephthalate (rPET) blends is crucial for the effective mechanical recycling of these commercial plastic wastes. This study investigates the reactive extrusion of PP/rPET blends using a dual compatibilizer system comprising maleic anhydride grafted polypropylene (PP-g-MA) and various glycidyl methacrylate (GMA)-based compatibilizers. The effects of backbone structure and reactive group on the morphological, mechanical, and thermal characteristics were systematically studied. This study sheds light on the effective compatibilization mechanisms using characterization methods such as Fourier Transform Infrared Spectroscopy (FTIR) and morphological analyses (SEM). The results indicate that GMA-based compatibilizers play a bridging role between rPET and PP-g-MA, resulting in improved compatibility between the blend components. A combination of 3 phr PP-g-MA and 3 phr ethylene-methyl acrylate glycidyl methacrylate terpolymer (EMA-GMA) significantly improves interfacial adhesion, leading to synergistic enhancements of mechanical performance of the blend, up to 217% and 116% increases in elongation at break and impact strength, respectively, compared to the uncompatibilized sample. Moreover, a significant improvement in onset temperature for degradation is observed for the dual compatibilized sample, with 40 °C and 33 °C increases in onset temperature relative to the uncompatibilized and the single compatibilized samples. These findings underscore the immense potential of tailored multi-component compatibilizer systems for upgrading recycled plastic waste materials.

## 1. Introduction

Polymers are renowned for their lightweight, cost-effectiveness, and superior processability and are used in a broad range of applications [1,2,3]. Particularly, the packaging industry has emerged as a major sector within the global economy, accounting for approximately 42% of the total plastic production [4,5]. Among the various thermoplastics, poly (ethylene terephthalate) (PET) is extensively used in products such as bottles, food containers, and films due to its excellent physical, chemical, and mechanical properties [6]. However, its high consumption has resulted in a substantial amount of PET waste, leading to significant environmental and economic challenges [7,8]. The increasing trend of plastic waste production has made recycling of PET vital to mitigate environmental repercussions and promote resource sustainability [9,10]. Polymer blending is an efficient and convenient strategy for mechanical recycling, enabling the development of materials with enhanced performance and new functionalities [6,11,12].

Polypropylene (PP), recognized as a preferable polymer for blending, is frequently selected to be combined with PET for various applications, including packaging, automotive, textile, and cosmetics [12,13,14,15,16]. This blending strategy integrates the remarkable properties of PP, such as chemical and heat resistance, ductility, and moisture barrier capabilities, with the superior oxygen barrier and high mechanical properties (high elastic modulus and mechanical strength) of PET [4,17,18]. Notably, the extensive use of both polymers, particularly in the packaging industry, typically results in the combination of PP and PET being present in the recycling stream due to incomplete separation and sorting processes [17,19]. However, the differences in chemical nature and polarity between PP and recycled PET (rPET) lead to thermodynamic immiscibility [20,21,22]. This incompatibility results in very low interfacial adhesion and phase separation between two components, adversely affecting the blend’s mechanical performance [12,23,24].

The incorporation of a compatibilizer, both reactive and non-reactive, has been shown to enhance the compatibility between components in such immiscible blends [25,26,27,28]. In recent years, numerous studies have investigated the effect of different compatibilizers with various backbone and reactive groups on the physical and mechanical properties of blends [3,29,30]. The most common compatibilizers reported for PP/PET blends contain reactive functional groups like maleic anhydride (MA) [21,31,32], acrylic acid (AA) [33], and glycidyl methacrylate (GMA) [20,33,34,35]. Asgari and Masoomi [31] compared the effect of two grafted copolymers, including glycidyl methacrylate grafted polypropylene (PP-g-GMA) and maleic anhydride grafted polypropylene (PP-g-MA), on the mechanical properties of PP/PET blends. They observed an enhancement in both tensile and impact properties upon the addition of 10 phr compatibilizer to the PP/PET fiber composites, particularly for the PP-g-GMA-based sample. Wang et al. [12] reported that introducing polyolefin grafted maleic anhydride (POE-g-MA) into PP/PET blends and utilizing multi-flow vibration injection molding technology (MFVIM) optimizes the mechanical properties for samples containing 10 phr PET and 4 phr POE-g-MA, achieving yield strength and impact strength of 50.87 MPa and 13.71 kJ/m^2^, respectively. They attributed this enhancement to the formation of a shish-kebab crystalline and core-shell structure featuring an elastomeric shell and PET core. Coba-Daza et al. [20] studied the effect of varying concentrations of an ethylene-butyl acrylate glycidyl methacrylate terpolymer (PTW) compatibilizer on physico-chemical characteristics of a PP/PET 70/30 blend. They found the most significant improvement in mechanical properties belongs to the sample containing 1.5% PTW due to the localization of the compatibilizer at the interface [33].

Despite extensive studies on processing PP/PET and PP/rPET blends, the potential synergistic effect of combining different compatibilizers on bridging the interphase between the two polymers is still unexplored. Furthermore, the impact of the compatibilizer’s various molecular structures and reactive groups on the resulting mechanical and physical properties needs to be clarified. Therefore, in this research, a novel approach involving dual compatibilizer systems, namely PP-g-MA and various GMA-based compatibilizers, is employed to investigate the influence of dual compatibilizers and the resulting in situ graft copolymers on enhancing the PP/rPET blend’s performance. The primary aim is to characterize the morphological, mechanical, and thermal behaviors of the PP/rPET 80/20 blend compatibilized by different combinations. The best combination of compatibilizers is identified for enhanced performance, and comprehensive insights into the underlying compatibilization mechanisms responsible for the observed improvements are laid out. Accordingly, this study can contribute significantly to resolving the important issue of recycling waste material streams with similar incompatibility for industrial applications, providing more effective and sustainable material designs.

## 2. Experimental Sections

### 2.1. Materials

A commercial-grade polypropylene (PP, D115A) (Braskem, Philadelphia, PA, USA) with a melt flow rate (MFR) of 11 g/10 min (2.16 kg, 230 °C) was utilized as the polymer blend matrix. Recycled poly (ethylene terephthalate) (rPET clear flakes) (PT Langgeng Jaya Plastindo, Gresik, Indonesia) was used as the dispersed phase. Polypropylene grafted maleic anhydride (PP-g-MA, OREVAC^®^ 18751) (generously donated by SK Functional Polymer, Courbevoie, France), with MFR of 35 g/10 min (2.16 kg, 230 °C), was used as the primary compatibilizer in all samples. The secondary compatibilizer sets, based on the reactive epoxide functional group (glycidyl methacrylate), were also donated by SK Functional Polymer, France. Table 1 summarizes the various characteristics of the compatibilizers. For comparative analysis with rPET, commercially available virgin PET with an intrinsic viscosity of 0.8 dL/g was used.

### 2.2. Sample Preparation

The polymer blend samples were prepared by melt compounding using a Process 11 co-rotating twin-screw extruder (Thermo Fisher Scientific, Waltham, MA, USA) at a speed of 200 rpm. The temperature profile within the various zones of the extruder was set at 70 °C, 180 °C, 190 °C, 220 °C, 240 °C, 260 °C, and 265 °C, going from the feeding zone to the die. Prior to compounding, rPET and PP-g-MA were dried for 18 h at 80 °C in a vacuum oven and then pre-mixed with the desired composition. Then, the processed extrudate was fed into a pelletizer to produce pellets in the size ranging from 2 to 4 mm. The compositions and corresponding sample codes are listed in Table 2. It is worth mentioning that the composition of the blend is expressed in terms of parts per hundred resins (phr), which indicates the number of compatibilizers per 100 parts of the base PP/rPET blend.

### 2.3. Characterizations

#### 2.3.1. Fourier Transform Infrared Spectroscopy (FTIR)

IR spectroscopic measurements were conducted using a Cary 630 FTIR spectrometer (Agilent Technologies, Santa Clara, CA, USA) spanning wavenumber ranging from 4000 to 400 cm^−1^ with a resolution of 4 cm^−1^. The FTIR analyses were used to investigate the potential chemical reactions between compatibilized PP/rPET blend components during the melt blending process.

#### 2.3.2. Scanning Electron Microscopy (SEM)

The morphology of cryo-fractured surfaces was examined by Scanning Electron Microscopy (SEM) (FEI XL30, Hillsboro, OR, USA) at an accelerating voltage of 15 kV. To obtain better contrast between the two phases, the samples were etched with an acid solution containing sulphuric acid and phosphoric acid in a proportion 2:1 and potassium permanganate with 0.67% wt/v solution at room temperature for 7 h. The sample surfaces were gold-sputtered to prevent electrostatic charging during examination.

#### 2.3.3. Rheological Characterization

The rheological measurements were performed using an Anton-Paar MCR 302 rheometer (Anton Paar GmbH, Graz, Austria) equipped with a 25 mm diameter parallel plate fixture using a 0.5 mm gap size. Samples were compression molded into 25 mm discs using a Carver benchtop molding press (Wabash, IN, USA). Initially, the linear viscoelastic region (LVR) of the PP matrix was determined through a strain sweep test (Figure S1). Subsequently, oscillatory frequency sweep tests were conducted at angular frequencies ranging from 0.1 to 1000 rad/s at 265 °C and a small strain amplitude of 1%, which was within the LVR.

#### 2.3.4. Differential Scanning Calorimetry (DSC)

The thermal properties and non-isothermal crystallization behavior of samples were analyzed using a TA DSC Q100 (TA instruments, New Castle, DE, USA) under a nitrogen-controlled atmosphere at a flow rate of 20 mL/min. All samples, weighing between 5 and 10 mg, were initially heated from room temperature to 280 °C at a heating rate of 10 °C/min and held for 5 min to eliminate any previous thermal history. Subsequently, they were cooled down to −10 °C at a constant rate of 10 °C/min and finally reheated to 280 °C at the same rate of 10 °C/min. The degree of crystallinity ($\chi_{c}$) was calculated using the following equation:

(1) $$\chi_{c}=\frac{{∆H}_{m}}{{∆H}_{m}^{0}\varphi }\times 100$$

where ${∆H}_{m}$ and φ represent the measured fusion enthalpy and mass fraction of PP in the blend, respectively. ${∆H}_{m}^{0}$, the fusion enthalpy of 100% crystallized PP, is equal to 207 J/g [36].

#### 2.3.5. Thermogravimetric Analysis (TGA)

A thermogravimetric analyzer (TGA) (Model: Q500, TA Instruments, New Castle, DE, USA) was utilized to examine the effect of compatibilizers on the thermal stability of the PP/rPET polymer blend. All samples, with weights ranging from 5 to 10 mg, were heated from 30 °C to 850 °C at a rate of 10 °C/min under a nitrogen-controlled atmosphere with a flow rate of 50 mL/min.

#### 2.3.6. Mechanical Properties

Tensile measurements were performed employing a universal tensile testing machine (Model no. 5965, Instron, Norwood, MA, USA) at room temperature (23 °C) and a crosshead speed of 5 mm/min. Five dumbbell-shaped samples were compression molded using a Carver benchtop molding press (Wabash, IN, USA) according to ASTM D638 [37] type IV standard’s specifications. Subsequently, the averaged value results were reported for each blend system.

Notched impact strength tests were carried out on molded specimens using an impact tester (Tinius Olsen, Horsham, PA, USA) according to ASTM D256 [38] at room temperature (23 °C). Bar-shaped samples with the dimensions of 63.5 mm × 12.7 mm × 3.2 mm were cut from compression-molded sheets, and then a 2.5 mm notch was made on the side. Tests were conducted using five samples of each blend and averaged.

## 3. Results and Discussion

### 3.1. In Situ Reactions between Components during the Mixing Process

**Figure 1:**
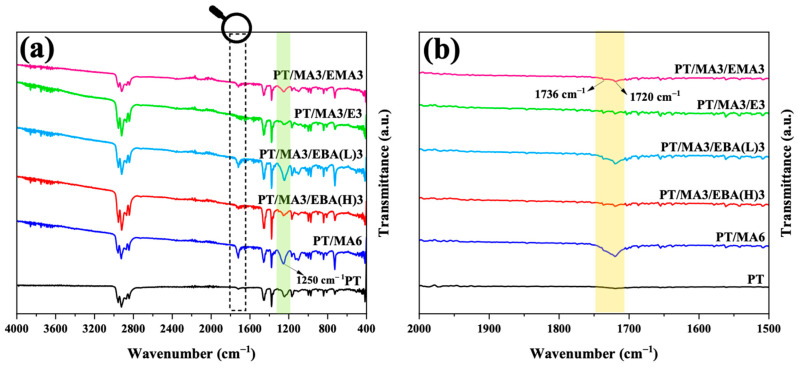
FTIR absorption spectra of the PT binary blend, the PT/MA6 blend containing single compatibilizer, and the blends with dual compatibilizers: (**a**) overall spectra for various blends and (**b**) magnification of a specific region in (**a**).

**Figure 2:**
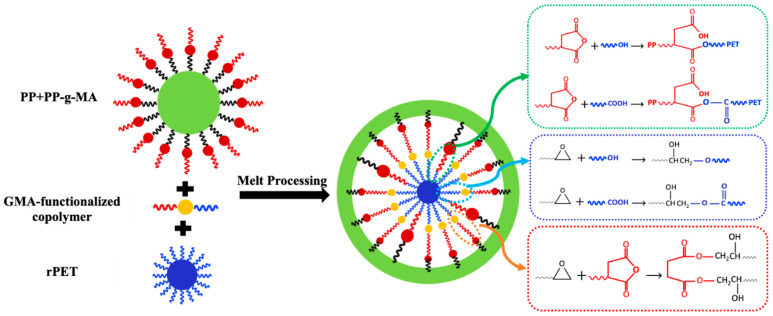
The mechanism of in situ reactions in melt blending of PP, rPET, PP-g-MA, and GMA-functionalized compatibilizers.

FTIR spectroscopy was employed to investigate the reactions between the –OH and –COOH functional end groups of rPET, the maleic anhydride of PP-g-MA, and epoxide groups of GMA-functionalized copolymers during the mixing process. Figure 1 shows the infrared spectra for the PP/rPET blend, the blend containing PP-g-MA compatibilizer, and four blends, each containing one of the secondary compatibilizers, i.e., PP-g-MA with one of the GMA-based copolymers. As can be observed in the uncompatibilized blend, the spectrum does not have a distinguishable peak at 1720 cm^−1^, corresponding to the (C=O) bond stretch of the carboxyl group. When PP-g-MA compatibilizer is introduced to the PT/MA6 sample, there is an evident increase in peak intensities at 1720 cm^−1^ and 1250 cm^−1^ related to the carboxyl group’s C=O and C-O stretches, respectively [1]. This highlights some significant microstructural changes for promoting better compatibility due to the chemical reaction between rPET end groups of carboxyl (–COOH) and/or hydroxyl (–OH) and MA of PP-g-MA. This results in the ring-opening of the MA group and forming carboxyl and excess ester (as conceptualized on the right side of Figure 2), being proven by a more pronounced peak at 1250 cm^−1^ attributed to the C–C–O bonding stretch of the saturated ester group [1]. It is noteworthy that the carboxyl end group of rPET is more reactive than its hydroxyl counterpart because of its inherent electron-withdrawing nature [11]. The weak IR absorbance at 1720 cm^−1^ wave number in the PT sample supports the hypothesis that PP and rPET are non-reactive [2,5].

When GMA-based compatibilizers are added as secondary compatibilizers, the FTIR peaks vary based on the GMA content. For the PT/MA3/EBA(H)3, PT/MA3/E3, and PT/MA3/EMA3 samples, which have a higher GMA content of 8%wt, there is a noticeable decrease in the intensity of the peak at 1720 cm^−1^ compared to the PT/MA6 sample. Moreover, the intensity of the peak at 1736 cm^−1^, corresponding to the (C=O) bonding stretch of the ester group, becomes distinguishable from 1720 cm^−1^ [20]. There are two possible scenarios when introducing the epoxy-based compatibilizer to the PP/rPET blend in the presence of PP-g-MA. Based on the two last illustrations on the right-hand side of Figure 2, the epoxide group of the GMA-based compatibilizer can react not only with the end groups of rPET but also with the MA group of PP-g-MA, resulting in ester formation in both cases. This is evidenced by the distinct peak at 1736 cm^−1^ [11]. Therefore, it is conceivable that GMA-based compatibilizers act as a bridge between PP-g-MA and rPET due to their potential to bind to both, mainly chemically to rPET and physically to PP, through MA and PP parts of PP-g-MA, respectively. However, for the PT/MA3/EBA(L)3 sample, which has lower amounts of GMA groups (5% wt), the peak at 1720 cm^−1^ shows a slight decrease compared to the PT/MA6 sample, and the peak at 1736 cm^−1^ appears as a shoulder. It can be concluded that the role of this second compatibilizer (EBA(L)) in reacting with both rPET and PP-g-MA is less pronounced than that of the samples processed with a GMA-based compatibilizer with higher epoxide group concentration.

Therefore, the compatibilization strategy between PP and rPET in the presence of the secondary GMA-based compatibilizer depends on the epoxide group concentration. Lower concentrations favor reactions between the maleic anhydride group of PP-g-MA and rPET end groups, while higher concentrations enable the GMA-based compatibilizer to react with both PP-g-MA and rPET.

Figure 2 demonstrates the proposed reaction-assisted compatibilization mechanism for the blends containing two compatibilizers. Accordingly, for all samples, the graft copolymers generated at the interface can form a dense shell of compatibilizer with high interfacial viscosity due to the reactive compatibilization, which can suppress the coalescence of dispersed droplets. This phenomenon, coalescence suppression, along with the reduction in the interfacial tension between PP and rPET, results in morphology stabilization and better compatibility with smaller droplet sizes of rPET [39].

### 3.2. Morphological Analysis

**Figure 3:**
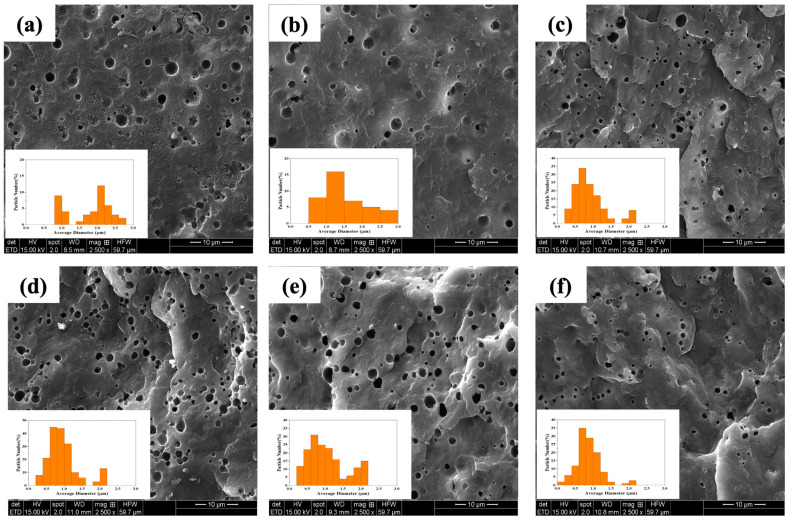
SEM images of cryo-fractured surfaces of (**a**) PT, (**b**) PT/MA6, (**c**) PT/MA3/EBA(H)3, (**d**) PT/MA3/EBA(L)3, (**e**) PT/MA3/E3, and (**f**) PT/MA3/EMA3, and inset is the particle size distribution of the blends.

Figure 3a–f illustrate the cryo-fractured surfaces of the PP/rPET binary blend, blend with one compatibilizer (PP-g-MA), and the other blends with dual compatibilizers (PP-g-MA and GMA-based compatibilizers), along with their respective average particle size distributions (as shown in the inset of each figure). The corresponding average size of the rPET phase is summarized in Table S1.

It is observed that in the uncompatibilized sample (Figure 3a), there are cracks throughout the sample, confirming the poor interfacial adhesion between the PP matrix and the rPET phase, which can lead to crack initiation, propagation, and failure. With the addition of PP-g-MA as a primary compatibilizer, the cracks disappear, and there is a reduction in the rPET particle size (Figure 3b), indicating that there is an improvement in compatibility between the two phases.

The incorporation of the secondary compatibilizers generally leads to a more uniform distribution and greater reduction in the rPET particle size (Figure 3c–f), suggesting that these compatibilizers play a significant role in enhancing the interfacial adhesion between two phases and suppressing coalescence [39]. Furthermore, the various backbone structures and GMA content of different compatibilizers influence the morphology of samples with dual compatibilizers. Specifically, the PT/MA3/EMA3 sample (shown in Figure 3f) displays a shift in the average particle size toward smaller diameters, as evidenced by the particle’s size distribution in Figure 3f and data in Table S1. This indicates a significant decrease in interfacial tension between the two phases accompanied by coalescence suppression [39,40]. Such a finer morphology enhances the final properties, such as mechanical properties, by improving stress transfer across the interface of the phases.

### 3.3. Rheological Behavior

**Figure 4:**
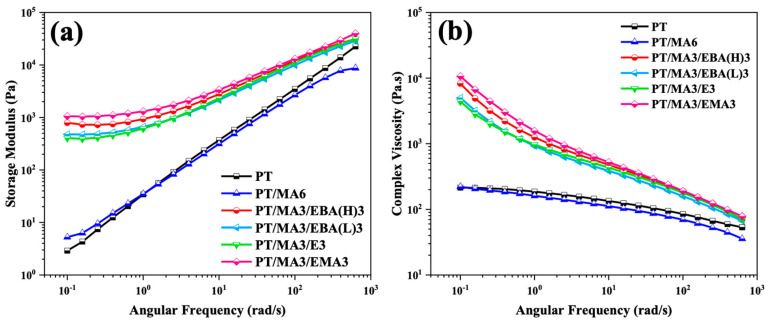
Storage modulus (**a**) and complex viscosity (**b**) versus angular frequency for the binary PP/rPET blend and the blends containing single and dual compatibilizers.

Rheology is a powerful tool for identifying polymer chain dynamics and the microstructural characteristics of polymer systems. In this study, the samples are investigated via frequency sweep tests conducted at small strain amplitudes in the linear viscoelastic region. Figure 4 illustrates the variation of storage modulus (G′) and complex viscosity (η*) as a function of angular frequency for the PP/rPET blend, the blend containing PP-g-MA compatibilizer, and blends containing dual compatibilizers.

As can be observed, compared to the uncompatibilized blend (PT) and the blend with one compatibilizer (PT/MA6), the addition of the secondary GMA-based compatibilizers increases the storage modulus and complex viscosity markedly, particularly in the low-frequency range. This modulus enhancement is indicative of network structure formation, highlighting the secondary compatibilizers’ pivotal role in improving the interfacial interaction between the PP and rPET phases [11,28]. Furthermore, the observed plateau at low frequencies for the storage modulus signifies a more elastic response, which can be attributed to the crosslinking reaction of the GMA-based compatibilizer and PP-g-MA and rPET, thus restricting the polymer chain dynamics [14]. A comparative analysis of samples containing dual compatibilizers reveals that the PT/MA3/EMA3 sample shows the highest storage modulus and complex viscosity values. This is explained by the presence of longer polymer chains, as evidenced by a lower MFI, enhanced chemical crosslinking density due to a higher concentration of GMA groups, and the presence of bulky groups or branches in the polymer structures, which restrict chain motion. Overall, these factors directly contribute to greater restriction of the chain dynamics and enhance the entanglement between the polymer chains in the system.

### 3.4. Crystallization Behavior Analysis

**Figure 5:**
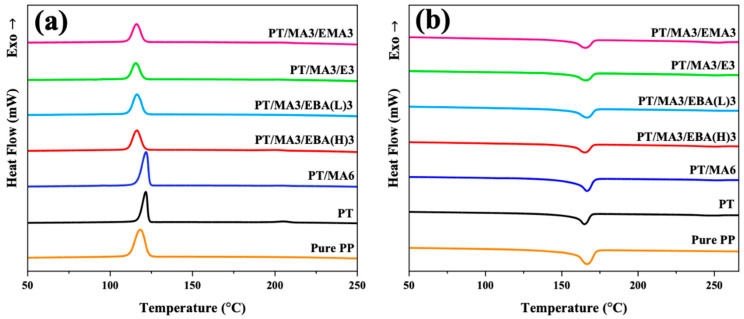
DSC cooling (**a**) and the second heating curves (**b**) of pure PP, the binary PP/rPET blend, and the blends containing single and dual compatibilizers.

In crystalline polymers, the crystallinity of the major component determines the physical and mechanical properties of the polymer blend. In this regard, the second heating and cooling processes of samples (Figure 5a,b) are evaluated to analyze the influence of the dual compatibilizer system on the crystallinity of the PP matrix and the ultimate performance of the blends. The relevant crystallization and melting properties are presented in Table 3. According to Figure 5a, the addition of rPET increases the PP crystallization temperature (T_c_) from 118.3 °C for pure PP to 121.6 °C for the PP/rPET blend. Additionally, the incorporation of rPET results in a narrower crystallization peak. The results imply that rPET droplets act as heterogenous nucleation (or simultaneous nucleation) sites for the PP phase, thereby forming PP crystallites within a narrower thickness range while imposing greater restrictions on chain dynamics. By introducing PP-g-MA to the binary PP/rPET blend, a slight increase in the crystallization temperature of PP is observed. It suggests that PP-g-MA’s reaction with rPET can enhance the nucleation effect by improving the distribution of nucleation sites. This behavior, along with an increase in crystallinity evidenced by the area under the crystallization peak (ΔH_c_), contributes to a more complete crystallization of PP [41].

The addition of the secondary compatibilizers significantly decreases the T_c_ and ΔH_c_ of the PP phase compared to the PT/MA6 sample. T_c_ and ΔH_c_ are important indicators of crystallite thickness or perfection and the proportion of polymeric chains with sufficient energy for the chain-folding process, respectively. The lower degree of crystallinity and reduced perfection in the dual-compatibilized samples can be due to the combination of two effects: (1) chemical reactions between the secondary compatibilizer, PP-g-MA and rPET (as discussed in Section 3.1), and (2) the presence of bulkier side groups or branches in the structure of GMA-based compatibilizers, which would suppress the crystallization kinetics [42]. Therefore, these factors reduce the interface nucleation effect and restrict the mobility of PP polymer chains to initiate forming an ordered crystalline structure. The reduction in ΔH_c_ further confirms the hindering role of the secondary compatibilizers in the formation of regular crystallites. It is worth mentioning that even though the structures of the secondary compatibilizers are different, the variation in T_c_ among samples with dual compatibilizers is not significant.

The second melting thermograms of PP/rPET blends—those without a compatibilizer, with one compatibilizer, and with dual compatibilizers—are presented in Figure 5b. It can be observed that the melting temperature (T_m_) of PP shows no significant change by incorporating PP-g-MA and GMA-based compatibilizers. However, the crystallization percentage of PP is noticeably affected. According to Table 3, the crystallization percentage of PP generally decreases by introducing the second compatibilizer compared to the PT/MA6. This can be attributed to the restricted movement of PP chains caused by the reactions between epoxide groups and HO-rPET-COOH and PP-g-MA. This reduction in crystallization percentage is further influenced by the GMA content and structure attributes of the secondary compatibilizer [43]. The sample with lower GMA content (PT/MA3/EBA(L)3) exhibits the highest crystallinity among all the samples containing dual compatibilizers. This can be related to the lower extent of in situ grafting reactions in this sample, which allows for chains to move more freely during chain packing. By evaluating the compatibilizers with various structures, the order of crystallization percentages is observed as the following order: PT/MA3/EBA(H)3 < PT/MA3/EMA3 < PT/MA3/E3. These results can be explained by the presence of different bulky groups or branches within the polymer structures. Consequently, the incorporation of pronounced bulky groups or branches, such as EBA, impedes the ability of PP chains to rearrange into a crystalline form.

### 3.5. Thermal Behaviour Analysis

**Figure 6:**
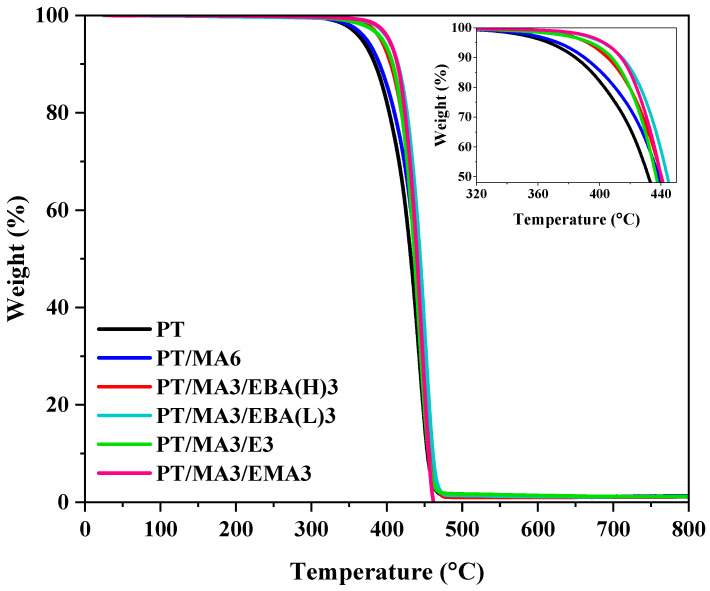
TGA curves of the binary PP/rPET blend and the blends containing single and dual compatibilizers.

TGA analysis was conducted to evaluate the thermal stability of the PP/rPET binary blend, the blend with one compatibilizer, and the other blends with two compatibilizers. Figure 6 presents the TGA thermograms of the aforementioned sample sets. The TGA data, including initial degradation temperature (T_onset_), the temperature at 10% weight loss (T_10_), and the temperature at 50% weight loss (T_50_), are presented in Table 4. It can be seen that the thermal degradation of the PP/rPET blend initiates at 367 °C, the earliest onset temperature observed across all examined samples. This can be attributed to the decreased molecular weight of rPET during the previous lifecycle and its poor interfacial adhesion to the PP phase. In fact, the presence of chain scission sites in the rPET structure, arising from prolonged aging, can act as starting points for thermal degradation, leading to a reduction in the activation energy required for initiating the degradation process [21,44]. The lower T_10_ of this sample compared to other samples agrees well with other results and our hypothesis.

The addition of PP-g-MA as the primary compatibilizer results in a slight increase in the initial degradation temperature of the blend, enhancing thermal stability. This increase can be attributed to the improved compatibility between PP and rPET phases, facilitated by the reaction of MA groups in PP-g-MA with functional end groups of rPET [3,45]. In the PP/rPET/PP-g-MA blend, the addition of the secondary compatibilizer, regardless of its structure and GMA content, leads to a significant improvement in thermal stability. This is evident by higher T_onset_ and T_10_ values. For instance, the T_onset_ of the best sample (PT/MA3/EMA3) has increased by 40 °C and 33 °C compared to the uncompatibilized sample and the sample with single compatibilizers (the same comparison can be made for T_10_ with 30 °C and 24 °C increases), respectively. These results underscore the crucial role of GMA-based compatibilizers in enhancing the interfacial adhesion between the PP and rPET phases, by which chain mobility is suppressed effectively and degradation resistance is reinforced [46].

### 3.6. Mechanical Properties and Toughening Mechanisms

**Figure 7:**
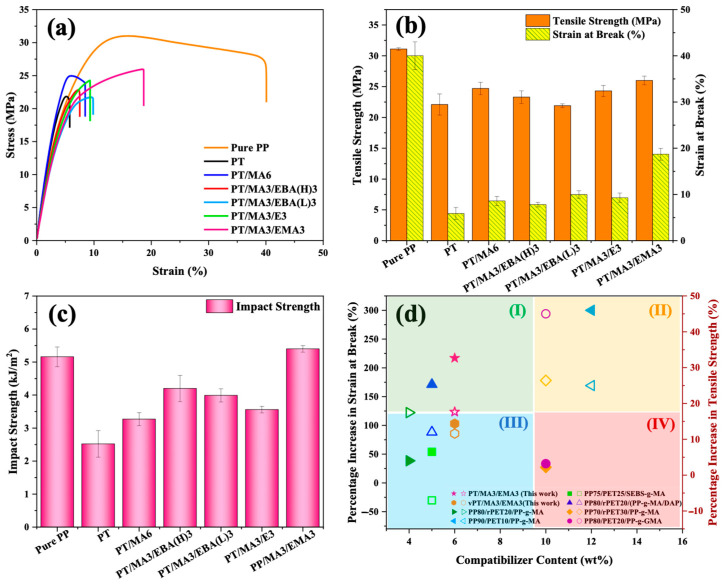
Mechanical properties of pure PP, the binary PP/rPET blend, and the blends containing single and dual compatibilizers: (**a**) tensile stress–strain curves, (**b**) tensile strength and elongation at break, (**c**) notched Izod impact strength, and (**d**) comparison of the results of the current study with the available literature data (filled symbols represent strain at break and hollow symbols represent tensile strength).

The effect of various compatibilizer combinations on the mechanical properties of PP/rPET blends was investigated via tensile and impact testing. Figure 7a presents the stress–strain curves, Figure 7b shows their corresponding results, including tensile strength and elongation at break, and Figure 7c shows notched Izod impact strength results of pure PP, uncompatibilized PP/rPET, and PP/rPET modified samples with single and dual compatibilizer systems. It can be observed that incorporating rPET into pure PP decreases its tensile properties and, more notably, the elongation at break. This behavior confirms the previous findings regarding the poor compatibility between PP and rPET components, where stress cannot be transferred effectively across the interface [2]. The introduction of the primary compatibilizer, PP-g-MA, into the binary blend results in improved tensile properties. This enhancement is attributed to the ability of PP-g-MA to form chemical bonds at the interface, thereby improving interfacial adhesion and facilitating smoother stress transfer between PP and rPET phases [47,48].

To evaluate the impact of incorporating dual compatibilizers on mechanical properties, the significance of factors such as chain length (indicated by MFI), acrylic ester group content, and GMA content varies depending on the specific mechanical property being examined. For tensile strength, a longer chain length (or a lower MFI) and a higher GMA content are beneficial. These factors collectively enhance chemical and physical crosslinking, promoting interfacial adhesion for a more effective load transfer [49]. As a result, a sequential enhancement in tensile strength is observed among the samples, as follows: PT/MA3/EBA(L)3 < PT/MA3/EBA(H)3 < PT/MA3/E3 < PT/MA3/EMA3. The PT/MA3/EMA3 sample achieves the highest tensile strength of 25.9 MPA, indicating superior chain interactions and the effectiveness of the MA3/EMA3 dual compatibilizer system.

Most samples showed comparable performance for elongation at break. However, the PT/MA3/EMA3 sample was of a notable exception, exhibiting a substantial increase in elongation at break, 217% higher than the uncompatibilized control (PT) and 116% higher than the sample containing the single compatibilizer (PT/MA6). This remarkable result suggests a synergistic effect of a lower MFI and high contents of acrylic ester and GMA, leading to the formation of an entanglement-enabled matrix. Along with sufficient crosslinking and improved adhesion, the reduced crystallinity (X_c_) in PT/MA3/EMA3 confirms greater chain mobility and flexibility, enabling the material to undergo more deformation before breaking [50,51]. The stress–strain curve presented in Figure S2 confirms the synergistic effect in blends with dual compatibilizers compared to the samples containing one compatibilizer.

Figure 7c shows the notched Izod impact strengths of PP/rPET blends with various compatibilizers, and it is clearly seen that the incorporation of dual compatibilizers increases the material’s toughness. This behavior demonstrates that the second compatibilizer can act as a bridge between PP/PP-g-MA and rPET, leading to better energy dissipation within the material during the impact test. Among the samples with dual compatibilizers, the PT/MA3/EMA3 sample emerges as the best, highlighting the balanced interplay of molecular chain length, the inherent flexibility of acrylic ester groups, and the reactive chemical functionality characteristic of GMA [52]. These findings are consistent with tensile results and crystallization behavior, where a lower degree of crystallinity with less perfection, accompanied by the presence of a more integrated network in the dual-compatibilized systems, enhances energy dissipation during the impact test.

A comparative analysis of the percentage increase in elongation at break and tensile strength for the best compatibilizer combinations in this study, namely PT/MA3/EMA3 and vPT/MA3/EMA3, relative to their respective binary blends and the available literature data, is illustrated in Figure 7d and Table S2. It can be seen that the PT/MA3/EMA3 sample falls in quadrant (I), which provides the highest properties’ enhancement with minimum compatibilizer addition. This illustrates the significant strides of this study in developing an effective strategy for recycling PP/rPET.

## 4. Conclusions

In this study, the efficiency of a dual compatibilizer system comprising PP-g-MA and various GMA-based compatibilizers was investigated to enhance the interfacial adhesion of PP/rPET binary blends. The influence of variations in the backbone structure and GMA content of the secondary compatibilizers was evaluated based on the blend’s microstructure and rheological, thermal, and mechanical properties. The key results are listed as follows:

- FTIR and morphological analyses demonstrated that GMA-based compatibilizers acted as a bridging agent between the PP/PP-g-MA and rPET phases by forming graft copolymers at the interface via in situ reactions during melt blending.
- The results reveal that compatibilizers with higher GMA content exhibit more extensive in situ grafting reactions, leading to improved compatibility between the blend components.
- DSC analysis of the blends with dual compatibilizers indicates that the addition of GMA-based compatibilizers alters the crystallization behavior, leading to a reduction in crystallization temperature, crystallization enthalpy, and degree of crystallinity. These observations confirm the role of the secondary compatibilizers in suppressing the crystallization process.
- Notably, for the PT/MA3/EMA3 sample, mechanical properties such as elongation at break, tensile strength, and notched Izod impact strength increased by 217%, 17.7%, and 114.3%, respectively, compared to the uncompatibilized sample. The enhanced performance can be attributed to the high GMA content and its reactions with the functional end groups of rPET and MA, which improved energy transfer. Additionally, the longer chain length and presence of flexible acrylic ester groups in the EMA compatibilizer contributed to increased mechanical properties.
- The same considerable improvement in thermal properties in terms of onset temperature for degradation is observed for the PT/MA3/EMA3 sample, with 40 °C and 33 °C increases compared to the uncompatibilized and the single compatibilized samples.
- This study highlights the potential of tailored dual compatibilizer systems to upgrade PP/rPET blends. The insights gained from this research can be used to develop more effective material designs for recycling thermodynamically incompatible plastic waste streams.

## Figures and Tables

**Table 1:** Characteristics and codes for the second compatibilizers used in this study.

Compatibilizer	Code	Grade	Acrylate Content (%wt)	GMA Content (%wt)	MFI (g/10 min)
Ethylene-butyl acrylate-glycidyl methacrylate terpolymer	EBA(H)	LOTADER AX8700	25	8	9
	EBA(L)	LOTADER AX8750	25	5	12
Ethylene-glycidyl methacrylate copolymer	E	LOTADER AX8840	0	8	5
Ethylene-methyl acrylate glycidyl methacrylate terpolymer	EMA	LOTADER AX8900	24	8	6

**Table 2:** Composition and code for different samples.

Sample Code	PP (phr)	vPET (phr)	rPET (phr)	PP-g-MA (phr)	2nd Compatibilizer (phr) *			
					1	2	3	4
Pure PP	100	0	0	0	0	0	0	0
vPT	80	20	0	0	0	0	0	0
PT	80	0	20	0	0	0	0	0
PT/MA6	80	0	20	6	0	0	0	0
PT/MA3/EBA(H)3	80	0	20	3	3	0	0	0
PT/MA3/EBA(L)3	80	0	20	3	0	3	0	0
PT/MA3/E3	80	0	20	3	0	0	3	0
PT/MA3/EMA3	80	0	20	3	0	0	0	3

* 1: EBA(H), 2: EBA(L), 3: E, 4: EMA.

**Table 3:** Non-isothermal crystallization parameters of pure PP, the binary PP/rPET blend, and the blends containing single and dual compatibilizers.

Sample	T_c_ (°C)	T_m_ (°C)	ΔH_c_ (J/g)	ΔH_m_ (J/g)	Xc (%)
Pure PP	118.3	166.7	110.0	92.0	44.4
PT	121.6	164.9	78.9	79.0	47.7
PT/MA6	121.8	166.6	91.3	82.6	49.8
PT/MA3/EBA(H)3	116.7	165.2	73.0	60.9	36.8
PT/MA3/EBA(L)3	116.3	166.7	87.2	76.6	46.3
PT/MA3/E3	115.6	165.9	76.3	67.2	40.6
PT/MA3/EMA3	115.9	165.6	75.9	61.5	37.2

**Table 4:** TGA results of the binary PP/rPET blend and the blends containing single and dual compatibilizers.

Sample	T_onset_ (°C)	T_10_ (°C)	T_50_ (°C)	T_max_ (°C)
PT	367	384	431	444
PT/MA6	374	390	438	449
PT/MA3/EBA-(H)3	390	405	440	444
PT/MA3/EBA-(L)3	407	415	444	451
PT/MA3/E3	403	408	437	440
PT/MA3/EMA3	407	414	440	446

## Data Availability

Data are contained within the article and Supplementary Materials.

## Supplementary Materials

The following supporting information can be downloaded at: https://www.mdpi.com/article/10.3390/polym16172439/s1, Table S1. Average size of rPET in the binary PP/rPET blend, the blend containing single and dual compatibilizers. Table S2. Comparison of the results of current study with available literature data. Figure S1. Dynamic strain sweep test for PP/rPET blend at frequency 1 rad/s. Figure S2. Tensile stress-strain curves for the uncompatibilized PP/rPET blend, the PP/rPET blend containing single compatibilizer (PT/MA6 and PT/EMA6), and the PP/rPET blend with dual compatibilizers. It includes additional information about morphological, rheological, and mechanical properties.
