# Supplementary material for: A Promising Recycling Strategy via Processing Polypropylene/Recycled Poly(ethylene terephthalate): Reactive Extrusion Using Dual Compatibilizers
Source: Polymers (Basel). 2024 Aug 28;16(17):2439. doi: 10.3390/polym16172439 (PMC11398067; doi:10.3390/polym16172439)
Supplement: Supplementary file 1 [file polymers-16-02439-s001.zip › polymers-3165617-supplementary.pdf]

polymers

MDPI

A promising recycling strategy via processing polypropylene/recycled poly (ethylene terephthalate): reactive extrusion using dual compatibilizers

Fatemeh Morshedi Dehaghi, Mohammad Aberoumand and Uttandaraman Sundararaj *

Department of Chemical and Petroleum Engineering, University of Calgary, Calgary, AB T2L1Y6, Canada
* Correspondence: authors: Prof. Uttandaraman Sundararaj; E-mail address: ut@ucalgary.ca, Department of Chemical and Petroleum Engineering, University of Calgary, Calgary, Alberta T2N 1N4, Canada

- ***Morphological analysis: Average size of rPET partciles***

Table S1. Average size of rPET in the binary PP/rPET blend, the blend containing single and dual compatibilizers.

sample	$\bar{d}_w$(μm)
**PT**	2.01
**PT/MA6**	1.00
**PT/MA3/EBA(H)3**	0.89
**PT/MA3/EBA(L)3**	0.94
**PT/MA3/E3**	0.99
**PT/MA3/EMA3**	0.87

- ***Rheological properties: Determination of linear viscoelastic region in binary PP/rPET blend as a reference sample.***

Figure S1 illustrates the results of a strain sweep test conducted on a PP/rPET blend, used as a reference sample to determine the linear viscoelastic region. This test was carried out over a strain amplitude range of 0.01 to 1000% at 265 °C and an angular frequency of 1 rad/s. It can be observed that the sample remains within the linear viscoelastic region across the entire strain range. Therefore, a strain amplitude of 1% was selected to avoid the nonlinear response when adding other components.

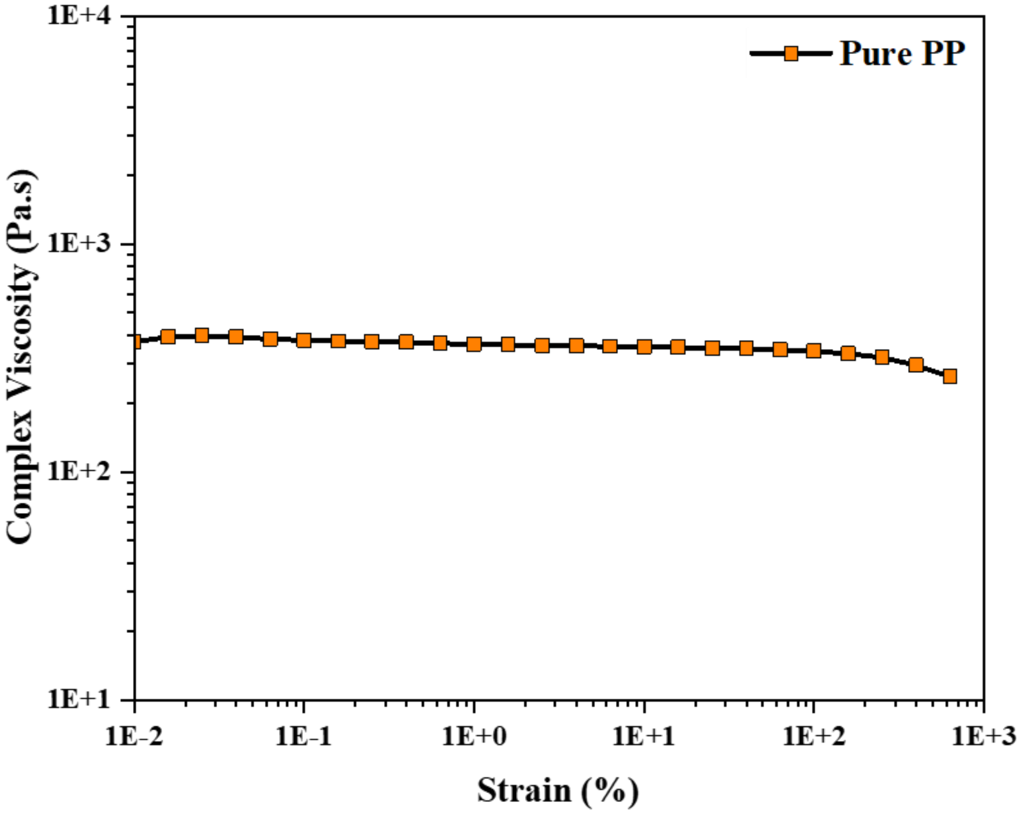

Figure S1. Dynamic strain sweep test for PP/rPET blend at frequency 1 rad/s.

- ***Mechanical properties: investigation the synergistic effect of dual compatibilizers***

Figure S2 presents the stress-strain curves for the uncompatibilized PP/rPET blend, the PP/rPET blend was modified using two different compatibilizers: one with PP-g-MA (referred to as PT/MA6) and the other with EMA-GMA (referred to as PT/EMA6), and the PP/rPET blend with dual compatibilizers. As can be seen, the PP/rPET blend sample containing dual compatibilizers exhibits a synergistic effect in tensile properties, including elongation at break and tensile strength.

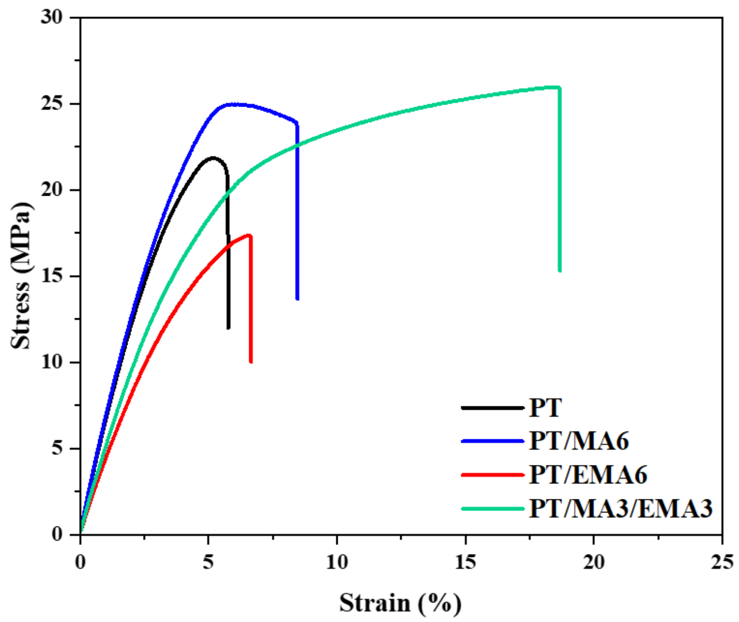

Figure S2. Tensile stress-strain curves for the uncompatibilized PP/rPET blend, the PP/rPET blend containing single compatibilizer (PT/MA6 and PT/EMA6), and the PP/rPET blend with dual compatibilizers.

- ***Comparison of present study with the literature***

Table S2. Comparison of the results of current study with available literature data.

sample	Compatibilizer content	Elongation increase	Tensile strength increase	Ref.
PP80/rPET20/PP-g-MA	4	38.5	17.4	[1]
PP70/rPET30/PP-g-MA	10	27.1	26.4	[2]
PP80/rPET20/(PP-g-MA/DAP)	5	171.0	12.0	[3]
PP80/PET20/PP-g-GMA	10	33.3	45.0	[4]
PP90/PET10/PP-g-MA	12	300.0	25.0	[5]
PP75/PET25/SEBS-g-MA	5	53.8	-7.0	[6]
PT/MA3/EMA3	6	217.0	17.7	This work
vPT/MA3/EMA3	6	103.0	11.6	This work

References

1. Akshaya, E.M.; Palaniappan, R.; Sowmya, C.F.; Rasana, N.; Jayanarayanan, K. Properties of Blends from Polypropylene and Recycled Polyethylene Terephthalate Using a Compatibilizer. *Mater Today Proc* **2020**, *24*, 359–368, doi:10.1016/J.MATPR.2020.04.287.
2. Ekinci, A.; Öksüz, M.; Ates, M.; Aydin, I. Thermal and Mechanical Properties of Polypropylene/Post-Consumer Poly (Ethylene Terephthalate) Blends: Bottle-to-Bottle Recycling. *Journal of Polymer Research* **2022**, *29*, 1–13, doi:10.1007/S10965-022-03229-6/FIGURES/8.
3. Zhu, Y.; Liang, C.; Bo, Y.; Xu, S. Compatibilization of Polypropylene/Recycled Polyethylene Terephthalate Blends with Maleic Anhydride Grafted Polypropylene in the Presence of Diallyl Phthalate. *Journal of Polymer Research* **2015**, *22*, 1–12, doi:10.1007/S10965-014-0591-4/FIGURES/12.

4. Asgari, M.; Masoomi, M. Thermal and Impact Study of PP/PET Fibre Composites Compatibilized with Glycidyl Methacrylate and Maleic Anhydride. *Compos B Eng* **2012**, *43*, 1164–1170, doi:10.1016/J.COMPOSITESB.2011.11.035.
5. Zhao, C.; Zhao, J.; Mark, L.H.; Chen, Z.; Soltani, I.; Lee, P.C.; Park, C.B. A Novel Strategy of Implementing Coupling Agents in Micro/Nano-Fibrillated Composites. *Polymer (Guildf)* **2023**, *273*, 125837, doi:10.1016/J.POLYMER.2023.125837.
6. Van Kets, K.; Delva, L.; Ragaert, K. Structural Stabilizing Effect of SEBSgMAH on a PP-PET Blend for Multiple Mechanical Recycling. *Polym Degrad Stab* **2019**, *166*, 60–72, doi:10.1016/J.POLYMDEGRADSTAB.2019.05.012.
